# Beyond Nitrogen Removal: Emerging Contaminants and Environmental Risk in Anammox-Based Wastewater Treatment

**DOI:** 10.3390/microorganisms14102382

**Published:** 2026-10-18

**Authors:** Shuang Pei, Chao Pan, Dongdong Xu, Wenji Li, Wenda Chen, Ping Zheng, Meng Zhang

**Affiliations:** 1 State Key Laboratory of Environmental Criteria and Risk Assessment, Chinese Research Academy of Environmental Sciences, Beijing 100012, China; 18841274085@163.com; 2 Research Center of Environmental Pollution Control Engineering Technology, Chinese Research Academy of Environmental Sciences, Beijing 100012, China; 3 Australian Centre for Water and Environmental Biotechnology (ACWEB, Formerly AWMC), The University of Queensland, St. Lucia, QLD 4072, Australia; dongdong.xu@uq.edu.au; 4 Nanyang Environment and Water Research Institute, Nanyang Technological University, Singapore 637141, Singapore; wenji.li@ntu.edu.sg; 5 Centre for Mined Land Rehabilitation, Sustainable Minerals Institute, The University of Queensland, Brisbane, QLD 4072, Australia; wenda.chen@uq.edu.au; 6 Department of Environmental Engineering, College of Environmental & Resource Sciences, Zhejiang University, Hangzhou 310058, China; pzheng@zju.edu.cn (P.Z.); zhangm_environment@zju.edu.cn (M.Z.)

**Keywords:** anammox, wastewater treatment, emerging contaminants, environmental risk, process resilience

## Abstract

Anaerobic ammonium oxidation (anammox) is an energy-efficient and low-carbon process for nitrogen removal, but its wider application exposes anammox biomass to more complex contaminant mixtures. Existing reviews have examined contaminant inhibition and microbial responses, while their links to environmental risks have received less integrated attention. This review focuses on how representative chemical contaminants and particles affect anammox performance and microbial responses and how these changes may lead to environmental risks beyond nitrogen removal. Responses depend on contaminant type, concentration, exposure pattern, and biomass form (e.g., granules and biofilms). Common responses include contaminant retention in extracellular polymeric substances (EPS) and biomass, changes in granule or biofilm structure, cellular and redox stress, disruption of core anammox metabolism, and shifts in microbial interactions. These changes may lead to ammonium or nitrite leakage, risks related to antibiotic resistance genes and antibiotic-resistant bacteria, potential N_2_O emissions, and contaminant accumulation in solids. Recovery of nitrogen removal alone does not confirm recovery of microbial activity or a reduction in retained contaminant burdens. We therefore propose evaluating contaminant-stressed anammox systems across liquid, gas, and solid phases, using a source-exposure-response-risk-control framework for monitoring, risk control, and recovery. This framework supports the risk assessment and long-term management of anammox-based treatment.

## 1. Introduction

Anaerobic ammonium oxidation (anammox) is an energy-efficient process for biological nitrogen removal. Anammox bacteria convert ammonium and nitrite directly to dinitrogen gas under anoxic conditions, which reduces aeration demand, external carbon addition, and excess sludge production compared with conventional nitrification-denitrification [1]. Partial nitritation/anammox (PN/A) has been widely applied to ammonium-rich sidestreams [2], while partial denitrification/anammox (PD/A) and other anammox-based processes are also being explored for mainstream municipal wastewater, landfill leachate, industrial wastewater, and other complex streams [3,4,5]. These applications extend the role of anammox in low-carbon wastewater treatment, but they also expose anammox biomass to more variable operating conditions and a wider range of contaminants.

The contaminant profile varies with wastewater source. Municipal wastewater can contain pharmaceuticals, antibiotics, disinfectants, per- and polyfluoroalkyl substances (PFAS), microplastics, and nanoplastics [6,7]. Landfill leachate may contain high ammonium together with refractory organic matter, salinity, metals, PFAS, and other persistent compounds [5,6]. Hospital, pharmaceutical, livestock, and aquaculture wastewaters can introduce antibiotics, biocides, antibiotic-resistant bacteria (ARB), antibiotic resistance genes (ARGs), and mobile genetic elements [8,9]. Industrial high-ammonium streams may also contain metals, solvents, engineered nanoparticles, and other process-specific chemicals. In this review, emerging contaminants mainly include pharmaceuticals and antibiotics, PFAS, biocides, microplastics, nanoplastics, and engineered nanoparticles. Heavy metals are considered co-occurring toxic stressors, while ARGs and ARB are treated as biological risk factors. This review focuses on how contaminant exposure affects the anammox process and microbial responses and how these changes relate to environmental risk, rather than on exhaustive coverage of each contaminant class. These factors can occur together with operational stresses such as salinity, low temperature, organic loading, and nitrite accumulation, which may further change the response of anammox systems.

A growing number of studies have examined how these contaminants affect anammox performance and microbial characteristics. Reported responses include changes in extracellular polymeric substances (EPS), granule and biofilm structure, cell integrity, redox processes, key anammox reactions, functional genes, and microbial interactions [10,11,12,13,14,15]. Existing studies and reviews, however, have mainly focused on contaminant inhibition, toxicity mechanisms, or changes in nitrogen removal and specific anammox activity (SAA). These endpoints are essential for process evaluation, but they do not fully describe how environmental risk changes during contaminant exposure. Here, environmental risk refers to four treatment-related pathways: effluent nitrogen leakage, resistance-related and microbial risks, potential N_2_O emissions, and contaminant accumulation or release from retained biomass. Stable nitrogen removal under contaminant exposure does not exclude the presence of ARGs in the microbial community [16] or contaminant accumulation in biomass [17]. In contrast, severe inhibition may cause ammonium or nitrite leakage and disturb associated microbial pathways. Contaminants retained by EPS, granules, biofilms, or sorptive materials may also be transferred from the water phase to solids rather than being removed from the treatment system [17,18]. In addition, recovery after contaminant exposure may remain incomplete, particularly when contaminants have accumulated within the biomass [19,20]. Environmental risk therefore depends not only on whether a contaminant inhibits anammox, but also on the level and duration of exposure, as well as its retention, persistence, and transfer among liquid, gas, and solid phases.

This review examines anammox systems from this environmental risk perspective. It links wastewater sources and exposure conditions with process and microbial responses, and then evaluates effluent nitrogen leakage, resistance-related risks, potential N_2_O emissions, and contaminant retention or release from biomass. Particular attention is given to the mismatch between reactor performance and biological or environmental recovery. These links are integrated into a source-exposure-response-risk-control framework for monitoring, early warning, risk control, and stepwise recovery. The aim is to move beyond inhibition-centered assessment and provide a practical basis for evaluating anammox systems under complex contaminant exposure. This is a narrative review of representative studies selected for their relevance to exposure, anammox responses, and potential risk pathways. Here, process inhibition refers to reduced nitrogen conversion or anammox activity during exposure; biological recovery concerns the return of biomass function after stress reduction, while environmental risk reduction requires changes in the relevant measured risk indicators.

## 2. Anammox-Based Treatment Systems and Exposure Scenarios

In practical wastewater treatment, anammox is rarely operated as a standalone process. Because anammox bacteria require both ammonium and nitrite, they are usually coupled with nitrite-producing pathways and retained in granular sludge, biofilms, moving-bed carriers, membrane biofilms, or other attached-growth systems [2,3]. In PN/A, ammonia oxidation supplies nitrite either in the same reactor as anammox (single-stage PN/A) or in a separate upstream reactor (two-stage PN/A), and the process is widely used for ammonium-rich sidestreams. In a PD/A system, nitrite is produced from nitrate, and this process is increasingly studied for mainstream nitrogen removal [2,3,4]. These process configurations affect not only nitrogen conversion but also which microbial groups are exposed to contaminants and how long contaminants remain in contact with the biomass.

Municipal wastewater is an important exposure scenario for the broader application of anammox. Sidestream PN/A mainly treats concentrated reject water from sludge digestion, whereas mainstream systems target low-strength municipal wastewater containing ammonium. These streams may contain antibiotics, pharmaceuticals and personal care products, disinfectants, PFAS, microplastics, and nanoplastics [6,7]. Mainstream systems also operate under low temperature, low ammonium concentration, and variable organic carbon, which can modify the response to long-term trace contaminant exposure [4].

Other high-ammonium wastewaters often contain more complex contaminant mixtures. Landfill leachate may combine high ammonium with refractory organics, salinity, metals, PFAS, and other persistent compounds [5,6]. Hospital, pharmaceutical, livestock, and aquaculture wastewaters may contain antibiotics and biocides together with antibiotic-resistant bacteria (ARB), antibiotic resistance genes (ARGs), and mobile genetic elements [8,9]. Industrial high-ammonium streams may also contain metals, engineered nanoparticles, solvents, high salinity, and other process-specific chemicals [14,15]. The exposure profile of an anammox system therefore depends strongly on wastewater source.

Reactor configuration further shapes the exposure experienced by anammox biomass. In single-stage PN/A, ammonia-oxidizing bacteria (AOB) and anammox bacteria share the influent, although their local exposure may differ within granules or biofilms. In two-stage PN/A, the anammox reactor receives the effluent from the upstream nitritation stage, so its exposure may differ from that of the raw influent. In either configuration, contaminants may directly inhibit anammox bacteria or indirectly reduce nitrite supply by affecting AOB. In PD/A, effects on partial denitrifiers can change nitrate to nitrite conversion and alter nitrite availability [3,10]. Biomass form is also important. EPS in granules and biofilms can bind chemicals and particles, which may reduce short-term exposure but increase their retention in biomass [10,14]. Influent concentration alone therefore does not fully represent the exposure experienced by anammox cells. Aqueous concentrations describe reactor-water exposure, whereas the bioavailable fraction may differ because of sorption or complexation. Biomass-associated burdens and cumulative exposure also depend on retention, duration, and repeated inputs. These exposure differences among anammox configurations and biomass forms are summarized in Table 1.

**Figure 1:**
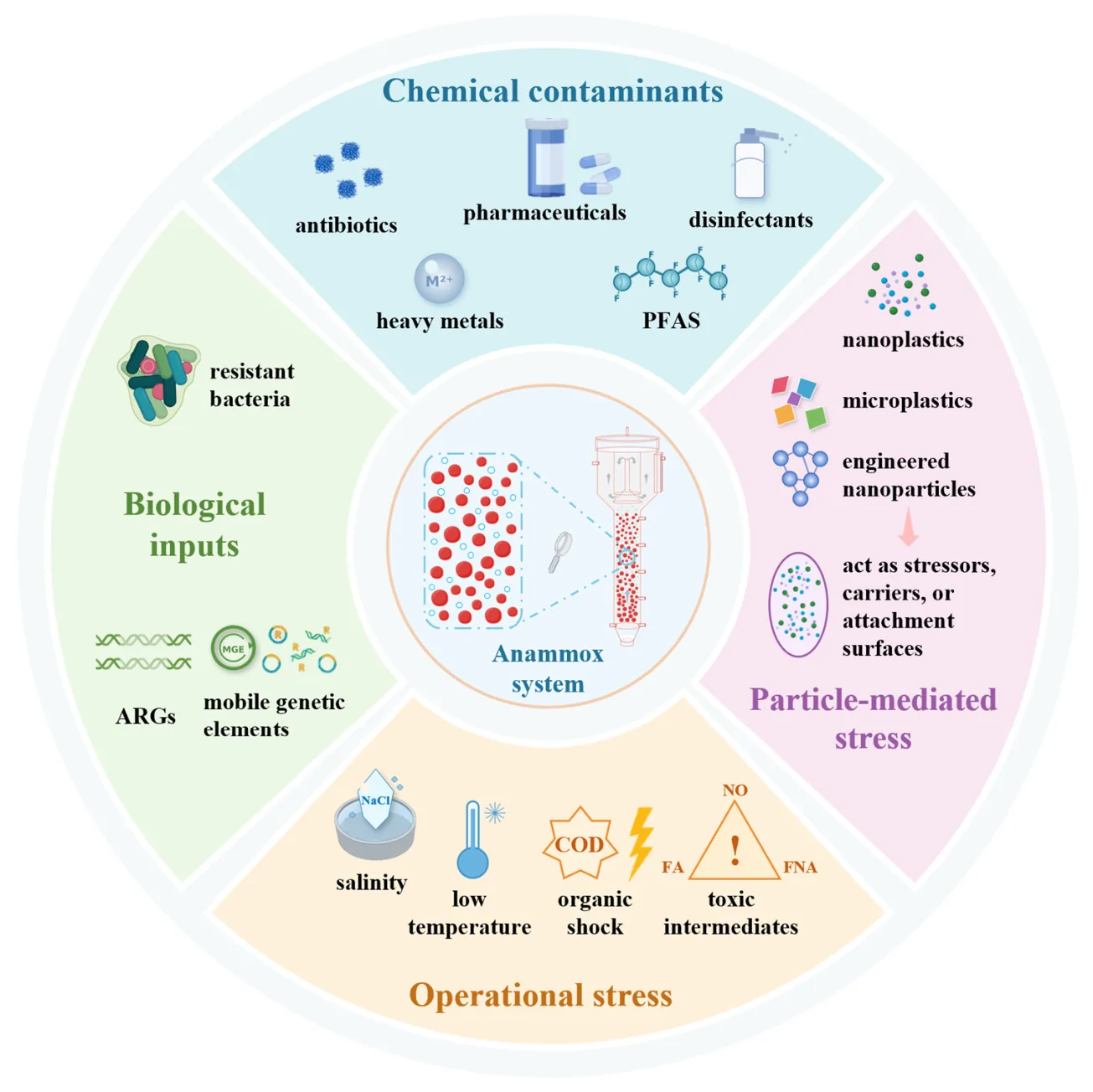
Major exposure related factors in anammox systems. Chemical contaminants, particle-mediated stress, and biological inputs represent the main external stressors, while operational conditions modify their effects on anammox biomass and the resulting environmental risks. Heavy metals are included as co-occurring toxic stressors rather than emerging contaminants.

Exposure assessment should consider contaminant concentration, exposure duration, mixtures, particle properties, biomass retention, and contaminant accumulation in sludge or biofilms [10,11,12,13,14,15]. Mixed contaminants may cause additive, synergistic, or antagonistic effects, depending on the contaminant combination and exposure conditions [13,21]. In real wastewater, dissolved organic matter, complexing agents, and competition for adsorption sites can change contaminant bioavailability [13,14,15]. Repeated or sequential exposure and long-term low-dose exposure may also produce responses different from those seen in short-term high-dose tests [17,22]. In this review, the main exposure related factors are organized into four groups (Figure 1). Chemical contaminants include antibiotics, pharmaceuticals, PFAS, and biocides, together with co-occurring toxicants such as heavy metals. Particle-mediated stress includes microplastics, nanoplastics, and engineered nanoparticles, which may act as stressors, carriers, or attachment surfaces. Biological inputs include ARB, ARGs, and mobile genetic elements. Operational stress includes low temperature, salinity, organic loading, nitrite accumulation, free ammonia, and free nitrous acid, which can modify contaminant responses. These groups can interact: particles may carry chemicals, chemical exposure may influence ARGs and ARB, and operational conditions can change both contaminant availability and microbial responses. This grouping is used to describe the main exposure conditions relevant to anammox systems rather than to classify all factors as emerging contaminants.

## 3. Effects of Contaminant Stress on Anammox Performance and Microbial Responses

The exposure scenarios described in Section 2 lead to different process and microbial responses. Contaminant stress can affect nitrogen removal, specific anammox activity (SAA), granule properties, functional genes, community composition, and resistance profiles [10,11,12,13,14,15]. This section compares these responses across the main contaminant groups relevant to anammox systems.

### 3.1. Antibiotics: Performance and Resistance-Related Responses

Antibiotic inhibition depends strongly on compound type, concentration, and exposure pattern. Long-term exposure to 2 mg/L oxytetracycline (OTC) reduced SAA by 81.3% after three weeks [23]. In a separate study using enriched anammox consortia, the system’s performance remained stable at 10 mg/L sulfadiazine (SDZ), whereas 1 mg/L chlortetracycline caused persistent inhibition without further treatment [16]. Exposure history also mattered: repeated SDZ shocks caused cumulative losses in SAA and total nitrogen removal efficiency (TNRE), while stepwise loading maintained TNRE above 85% at 50 mg/L [22]. Thus, concentration alone does not predict reactor response. As exposure duration, initial activity, biomass concentration, loading conditions, and acclimation history were not standardized across these studies, the results should not be used to directly rank antibiotic toxicity.

Performance changes are often accompanied by microbial responses. Under OTC exposure, heme c content and *hzs* abundance decreased by 50.1% and 29.7%, respectively [23]. Under SDZ and chlortetracycline stress, 18 ARG types and 94 subtypes were detected, with more than 20 genera identified as possible hosts [16]. *Candidatus *Brocadia was associated with 46.81% of SDZ-resistance genes and 38.63% of chlortetracycline-resistance genes, although these inferred associations do not prove host identity.

The main feature of antibiotic stress is therefore the possible separation between reactor performance and microbial condition. A reactor may maintain a high TNRE after acclimation while SAA or resistance-related profiles have already changed. Antibiotic effects should therefore be evaluated using both nitrogen-removal indicators and biomass-level responses, rather than judging tolerance from effluent performance alone.

### 3.2. Microplastics and Nanoplastics: Delayed Performance Loss and Biomass Responses

Plastic particles often show stronger effects during long-term exposure than during short-term tests. For polyethylene terephthalate microplastics (PET-MPs), exposure to 0.1–0.2 g/L produced no significant change in anammox performance, whereas 1.0 g/L reduced SAA by 16.2% [24]. A much longer exposure to 200 mg/L PET-MPs produced a different response: TNRE declined during the 282-day study, and approximately 87% of its initial value was recovered after three months [19]. A similar delayed response was observed for polystyrene nanoplastics (PS-NPs). Although 1–2 mg/L PS-NPs were tolerated during the early stage of operation, ammonium- and nitrite-based SAA values at day 225 were 44.5% and 57.6% lower than those at day 100. Strong inhibition became apparent when the accumulated PS-NP concentration in the biomass reached 118 mg/g VSS [17]. These results show that a low influent concentration does not necessarily mean a low long-term exposure when plastic particles are retained in the sludge.

Microplastics and nanoplastics differ in size and specific surface area, which can influence their interactions with anammox biomass. Larger MPs mainly interact with granule surfaces, whereas smaller NPs have a higher surface area per unit mass and may penetrate further into the EPS matrix. However, particle aggregation, aging, and adsorption of other contaminants can change these interactions, so particle size alone does not determine toxicity. Smaller particle size also does not necessarily indicate cellular uptake. For example, a recent study compared the effects of PET particles of 80 µm and 300 nm on anammox granular sludge [25]. Particle shape, surface properties, and whether concentrations were measured or nominal also affect cross-study comparisons.

The decline in performance is accompanied by changes in the physical and biological properties of anammox granules. Long-term PET- and PS-based particle exposure has been associated with changes in EPS, granule integrity, and microbial activity [17,19,25]. More recent work showed that PET micro- and nanoplastics can reduce nitrogen removal while causing surface cracking, structural loosening, and disintegration of anammox granules [25]. Changes in EPS and quorum-sensing-related processes were also observed, together with changes in genes involved in anammox and central metabolism [25]. These responses may be closely linked. Particle retention and changes in EPS can weaken granule structure and affect substrate transport, leading to reduced microbial activity. Lower metabolic activity may, in turn, limit EPS production and further weaken the granules. In addition, changes in quorum sensing may affect EPS regulation, as supported by improved EPS production following the addition of signaling molecules in the PET micro- and nanoplastic study [25]. These biomass responses help explain why anammox performance may remain relatively stable during early exposure but decline after particles have been retained for a longer period.

Other polymers have also shown different effects on anammox biomass. In a 40-day study, polyethylene microplastics (PE-MPs) at higher concentrations reduced EPS content and SAA [26]. Another study comparing polyvinyl chloride (PVC) and polybutylene succinate (PBS) microplastics found stronger inhibition of nitrogen removal under PVC exposure [27]. These findings further indicate that polymer type, together with particle size and exposure conditions, can influence the response of anammox systems.

Plastic-derived chemicals can also contribute to toxicity. Low-density polyethylene (LDPE) and polypropylene (PP) particles caused little direct inhibition under the tested conditions, whereas 31 and 37 compounds were tentatively identified in their leachates, respectively [28]. Dibutyl phthalate at 100 mg/L caused 29.4% inhibition after 5 h, followed by substantial recovery within 24 h [28]. Plastic particles and released chemicals should therefore be evaluated separately.

### 3.3. PFAS, Biocides, and Other Organic Contaminants: Performance and Microbial Responses

PFAS can inhibit anammox, although most quantitative evidence still comes from laboratory studies using relatively high concentrations. Increasing the perfluorooctanoic acid (PFOA) concentration from 5 to 50 mg/L reduced ammonium removal from 93.90% to 77.81% and nitrite removal from 98.60% to 77.96%, corresponding to decreases of 16.09 and 20.64 percentage points, respectively [29]. In a perfluorobutane sulfonate (PFBS) study, total nitrogen (TN) removal decreased from 95.60 ± 0.85% in the control to 85.89 ± 0.93% at the highest exposure level, a decrease of 9.71 percentage points [30]. These results show concentration-dependent responses but should not be interpreted as field thresholds.

PFAS exposure can also alter microbial characteristics before complete process failure. Under PFBS stress, *hzs* and *hdh* abundance decreased by 54.65% and 57.32%, respectively, while ARGs, mobile genetic elements, and potential pathogens increased [30]. These changes show that functional and microbial risk indicators can shift together with nitrogen removal.

Long-term information remains limited for biocides and many other organic micropollutants. Triclocarban (TCC) had little effect at 50 μg/L but caused clear inhibition and community changes at 200 μg/L [31]. Comparable long-term data are still scarce for many disinfectants, pharmaceuticals, and endocrine-disrupting compounds, especially under mixed and environmentally relevant exposure conditions.

### 3.4. Heavy Metals and Engineered Nanoparticles: Dose-Dependent Performance and Biomass Responses

Heavy metals are considered here as co-occurring toxic stressors rather than emerging contaminants. Their effects on anammox are strongly concentration dependent, and some metals can shift from beneficial at trace levels to inhibitory at higher concentrations. In batch tests, 1 mg/L Ni(II) increased SAA by 11.14%, whereas the fitted half-maximal inhibitory concentration (IC_50_) was 83.86 mg/L and inhibition became stronger with longer exposure [20]. A separate study reported a much lower batch IC_50_ of 14.64 mg/L for Ni(II), while an upflow anaerobic sludge blanket (UASB) reactor reached approximately 93% nitrogen removal after acclimation during a 150-day study at 10 mg/L Ni(II) [32]. Cu(II) showed a stronger inhibitory response: at 5 mg/L, nitrogen removal efficiency in the Cu(II)-exposed reactor without added biochar decreased from 99.0% to 25.48%, a decrease of 73.52 percentage points [33]. These differences show that a single batch inhibition value cannot fully predict the response of a continuously operated anammox reactor.

Engineered nanoparticles add another time-dependent component because they can be retained and accumulated in sludge. For ZnO nanoparticles, an acute IC_50_ of 84.7 mg/L was reported, and exposure to 200 mg/L caused about 80% inhibition [34]. During long-term exposure, 100 mg/L ZnO nanoparticles also inhibited anammox performance as the material accumulated in the biomass. After ZnO addition was stopped, recovery required about 120 d [34]. Thus, acute toxicity and long-term reactor response can differ substantially for the same material.

The common feature of metals and engineered nanoparticles is that their effects depend on both dose and exposure history. Biomass retention, acclimation, and accumulation can change the apparent tolerance of the reactor. For this reason, batch IC_50_ values are useful for toxicity screening but should be interpreted together with continuous-reactor performance and biomass-associated contaminant concentrations. Acute IC_50_ values, chronic inhibition levels, no-observed-effect levels, and recovery measurements represent different endpoints and should not be directly equated.

### 3.5. Cross-Study Patterns in Performance and Microbial Responses

Reported responses differ in test mode, concentration, exposure time, biomass form, and endpoint, so direct comparison based only on nominal concentration can be misleading. Table 2 summarizes representative studies including polymer type, particle size, exposure conditions, reactor type, and main effects where available.

Several patterns are consistent across studies. Short-term tolerance does not guarantee long-term stability, especially when contaminants accumulate in retained biomass. Reactor-level nitrogen removal can also remain stable while SAA, functional genes, granule properties, or resistance profiles have already changed. Contaminant effects should therefore be evaluated using both process performance and biomass-level responses, together with exposure duration, accumulation, and recovery.

## 4. Mechanisms of Contaminant Stress and Resilience

Section 3 described how contaminants change anammox performance and microbial responses. This section examines how these responses develop at different mechanistic levels. The mechanisms can be followed from the outside of the biomass inward: contaminants first interact with extracellular polymeric substances (EPS) and retained solids, then affect cell integrity and redox balance, interfere with core anammox reactions, change microbial interactions, and finally influence adaptation and recovery after exposure.

### 4.1. EPS and Biomass Structure Regulate Local Exposure

**Figure 2:**
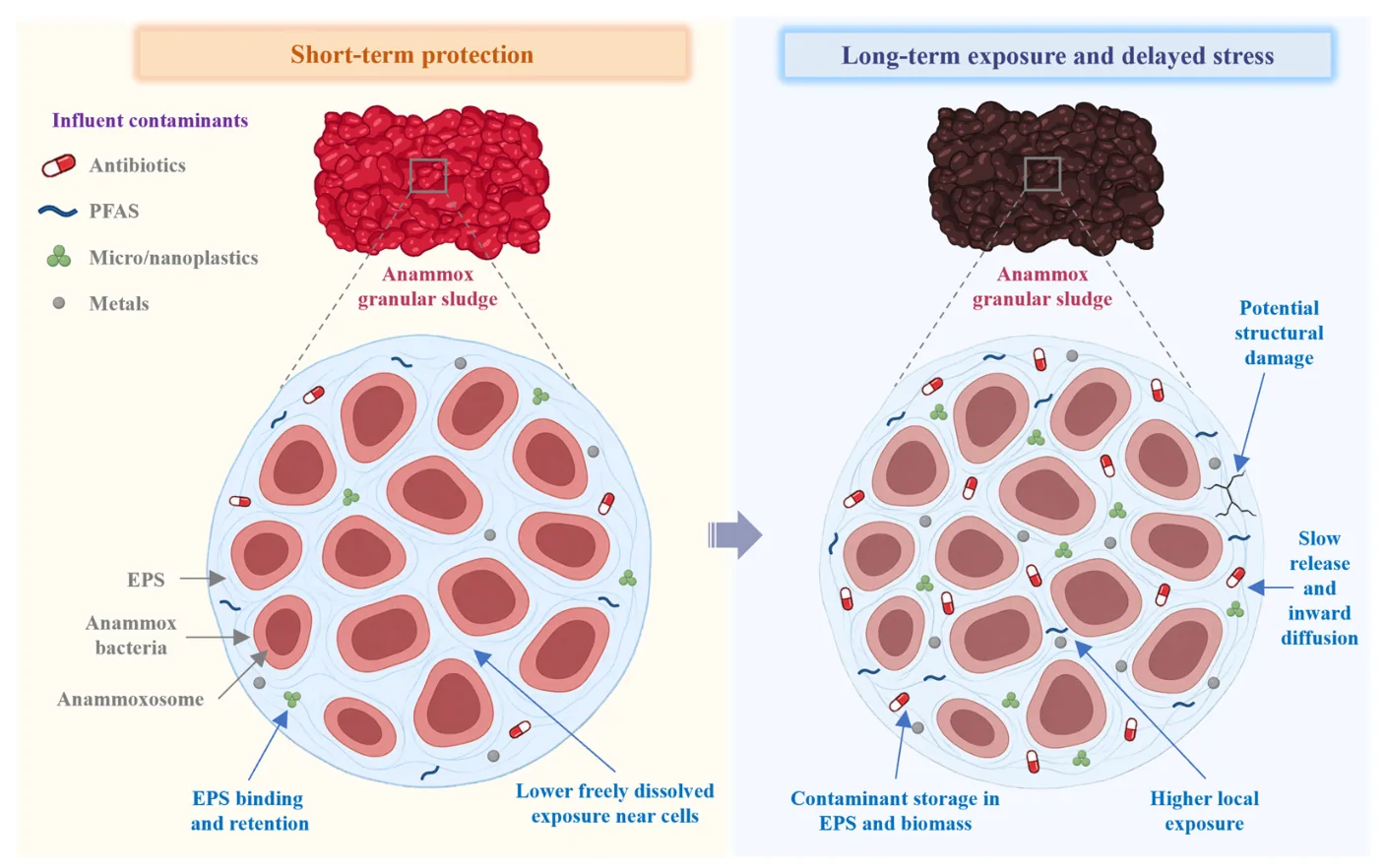
Conceptual schematic of the dual role of EPS in regulating contaminant exposure in anammox biomass. During short-term exposure, EPS can bind and retain contaminants, reducing the freely dissolved fraction reaching anammox cells. During prolonged exposure, retained contaminants may accumulate in EPS and biomass, undergo slow release and inward diffusion, increase local exposure, and contribute to structural damage and delayed inhibition. Retention is supported by reported measurements, whereas later inward diffusion and delayed damage are shown as possible pathways rather than a single established sequence.

EPS and biomass structure determine the local exposure experienced by anammox cells. Proteins, polysaccharides, humic-like compounds, and lipids form a reactive matrix around the cells. This matrix can bind dissolved contaminants and retain particles [10,12,13,14,15,35]. EPS can therefore reduce cellular exposure to freely dissolved contaminants during early contact, but it can also retain contaminants within granules and biofilms during prolonged exposure. This dual role is illustrated in Figure 2. Under mixed exposure, microplastics can adsorb co-occurring antibiotics, which may change their availability to anammox cells [13].

Changes in EPS can affect stress tolerance. Under PFOA exposure, the addition of 2 µmol/L N-hexanoyl-L-homoserine lactone (C6-HSL) increased ammonium removal from 79.7 ± 4.8% to 94.4 ± 4.3% and nitrite removal from 80.8 ± 3.8% to 97.1 ± 3.8% [36]. The improvement occurred with denser extracellular proteins, higher sludge hydrophobicity, and an increase in Planctomycetota from 30.2% to 49.5%. The parallel changes in EPS-related properties and nitrogen removal support a protective role of the biomass matrix, although the phylum-level shift is not a direct measure of anammox activity. Local exposure can also be reduced by sorptive materials. Iron-loaded biochar increased ammonium and nitrite removal under PFOA stress to 94.45 ± 5.1% and 95.9 ± 5.0%, while PFOA removal increased from 5.2% to 29.2 ± 4.3% [18]. Part of this removal may represent transfer from water to solids rather than degradation.

Protection can weaken when particles accumulate and damage the biomass. Long-term PS-NP exposure reduced EPS content and hydrophobicity, impaired mass transfer, and produced cracks in granules; strong inhibition appeared when biomass-associated PS-NPs reached 118 mg/g VSS [17]. PET-MPs also increased oxidative stress and weakened granule structure during prolonged exposure [19,24]. This helps explain why short-term tests can underestimate delayed structural damage.

### 4.2. Cell Damage and Oxidative Stress

Once contaminants pass through or disturb the outer biomass structure, cell integrity becomes an important control point. Damage to the cell envelope can change membrane permeability, substrate transport, and intracellular conditions. Oxidative stress can further disturb proteins, lipids, and redox processes that support anammox metabolism. These effects may occur before a clear decrease in reactor-level nitrogen removal is observed. Without oxidative biomarkers or other direct measurements, changes in granule structure or performance alone do not establish oxidative stress.

PET-MP exposure was accompanied by oxidative stress and loss of granule stability [19,24]. This structural weakening may increase cell-level exposure and is consistent with the delayed decline in TN removal observed during prolonged exposure.

Metal exposure provides clearer evidence that contaminants can move from the biomass surface into the cell. Under Ni(II) stress, longer exposure increased intracellular Ni accumulation while SAA decreased from 11.19 to 7.86 and then to 3.09 mg TN/(g VSS h) after 0, 12, and 48 h, respectively [20]. The increasing intracellular metal burden was accompanied by lower hydrazine dehydrogenase activity. This response suggests that once metal accumulation extends beyond surface binding, inhibition becomes more closely linked to intracellular metabolism and may become harder to reverse.

### 4.3. Electron Transfer and Core Anammox Metabolism

Cell stress affects reactor performance when it interferes with the core reactions of anammox. Nitrite reduction, hydrazine formation, and hydrazine oxidation depend on hydrazine synthase (HZS), hydrazine dehydrogenase/oxidoreductase (HDH/HZO), and heme-containing redox proteins [37,38]. Disturbance of these components can directly lower anammox activity.

Several studies connect performance loss with changes in these metabolic components. After three weeks at 2 mg/L OTC, SAA, heme c, and *hzs* abundance decreased by 81.3%, 50.1%, and 29.7%, respectively [23]. Under Ni(II) stress, intracellular accumulation coincided with lower hydrazine dehydrogenase activity and SAA [20]. PFBS exposure also reduced *hzs* and *hdh* abundance by 54.65% and 57.32%, together with lower TN removal [30]. Gene changes support disruption of anammox metabolism but do not by themselves prove direct enzyme inhibition. Gene abundance, transcript levels, protein levels, enzyme activity, and measured metabolic flux provide different kinds of evidence.

Intervention studies can provide support for proposed metabolic mechanisms, although several effects may occur simultaneously. Under PFOA stress, iron-loaded biochar increased *hzsB* and *hdh* abundance together with improved nitrogen removal [18]. Iron may support redox metabolism, but biochar also changes PFOA sorption, so the improvement cannot be attributed to electron transfer alone.

Molecular docking can identify possible enzyme targets but cannot prove inhibition in living cells. Molecular docking suggested potential interactions of SDZ with nitrite reductase [22] and PFBS with hydrazine dehydrogenase, with a calculated binding energy of about −8 kcal/mol [30]. Stronger evidence requires enzyme activity, intracellular exposure, transcript or protein data, and recovery measurements.

### 4.4. Microbial Interactions and Resistance Selection

Contaminants can also affect anammox indirectly by changing the microorganisms that supply or consume nitrite. In PN/A, inhibition of AOB can reduce nitrite production, whereas stronger inhibition of anammox bacteria can cause nitrite accumulation. In PD/A, changes in partial denitrifiers can reduce nitrate to nitrite conversion or favor complete denitrification. Reactor responses therefore depend partly on which microbial group is affected first [3,10].

Changes within the anammox community can further reduce nitrogen conversion. Under 50 mg/L PFOA, the relative abundance of *Candidatus *Brocadia decreased from 22.21% to 2.11% and *Candidatus *Kuenenia from 5.61% to 2.84%, together with lower ammonium and nitrite removal [29]. Because relative abundance can change as the abundance of other populations changes, absolute abundance or biomass-normalized gene copy numbers are needed to confirm actual biomass loss.

Resistance selection is another community-level response. Under SDZ and chlortetracycline stress, 18 ARG types and 94 subtypes were detected, with more than 20 genera associated with resistance genes [16]. Long-term OTC exposure increased *tetA* from 1.03% to 2.51%, while *tetB* and *tetC* approximately doubled [23]. PFBS exposure also increased ARGs, mobile genetic elements, and potential pathogens while lowering TN removal [30]. These changes indicate concurrent changes in process performance and microbial risk, but co-occurrence data do not prove host identity or horizontal gene transfer.

### 4.5. Adaptation and Recovery After Contaminant Exposure

Process tolerance, adaptation, and recovery describe different aspects of system resilience. Process tolerance is the ability to maintain function during exposure, adaptation is improved tolerance during continued or repeated exposure, and recovery is the return toward the pre-exposure state after stress is reduced. Effluent performance can recover before the biomass itself does.

Exposure history strongly affects adaptation. Stepwise SDZ loading maintained TNRE above 85% at 50 mg/L, whereas repeated shocks caused cumulative performance loss [22]. Biomass growth and protein-rich tightly bound EPS were associated with better performance during gradual loading, indicating that reactor-level adaptation can partly compensate for lower cell-specific activity.

Mixed contaminants may affect several of these mechanisms at the same time. For example, a study of PN/A biofilms showed that the combined effects of PFAS and a quaternary ammonium compound varied with exposure concentration, alongside changes in EPS properties and ammonium/nitrite transport [21]. This suggests that changes in local exposure and microbial function may jointly shape the response to mixtures. Under prolonged low-dose exposure, contaminants retained in biomass may accumulate over time, as observed for PS-NPs, leading to changes in EPS, granule stability, and anammox activity [17]. These findings help explain why short-term responses to individual contaminants may not fully reflect those under mixed or long-term exposure.

Recovery depends on the extent and location of contaminant accumulation. In the 282-day PET-MP study, TNRE recovered to approximately 87% of its initial value after three months [19]. In Ni(II) tests, the apparent IC_50_ increased from 10.83 to 30.89, 82.08, and 165.43 mg/L across four exposure cycles [20]. EDTA restored 93.25–98.49% of initial SAA after exposure to 2.5–25 mg/L Ni(II), but only 46.49–53.47% after 75–100 mg/L [20]. The effects associated with severe intracellular accumulation were therefore less reversible than those associated with moderate exposure. Recovery of nitrogen removal should not be taken as complete biological recovery if SAA, functional genes, granule structure, community composition, or ARG profiles remain altered.

The degree of recovery can be assessed relative to the pre-exposure state using nitrogen-removal performance and, where available, SAA, EPS, granule structure, functional genes, and microbial community composition. ARGs/ARB, N_2_O emissions, and retained contaminant residues can provide additional information on environmental risks during recovery when these indicators are measured.

### 4.6. Mechanistic Evidence Across Studies

Mechanistic conclusions should reflect the type of evidence available. Intracellular contaminant measurements and enzyme activity provide relatively direct evidence because they connect exposure with a physiological response. Measurements of membrane integrity, oxidative stress, ATP, transcripts, proteins, and metabolites can identify affected cellular processes. Functional gene abundance and community composition provide useful biological information. Co-occurrence networks can suggest possible microbial associations rather than direct ecological interactions or confirmed ARG hosts. Molecular docking can identify potential binding interactions, but such predictions require biochemical or physiological validation. For interpretation, we should distinguish direct physiological or biochemical measurements, molecular evidence, community associations, modeling or docking, and conceptual inference.

Overall, contaminant effects can be followed from retention in EPS and biomass to structural and cell stress, disruption of redox and core anammox metabolism, changes in microbial interactions, and variable recovery. This sequence links the process and microbial responses described in Section 3 with the environmental risks discussed in Section 5.

## 5. Environmental Risk Implications of Contaminant-Stressed Anammox Systems

Laboratory inhibition studies mainly characterize contaminant hazard. Environmental risk is more appropriately evaluated when exposure leads to adverse outputs, increased contaminant release, or contaminant transfer among the water, gas, and solid phases. The mechanisms discussed in Section 4 can lead to four main risk pathways. These include effluent nitrogen leakage, resistance-related and microbial risks, potential N_2_O emissions, and contaminant accumulation and release from retained biomass. This section evaluates these four risk pathways. These are potential pathways, not a quantitative assessment of downstream environmental impacts; measured nitrogen leakage and solid-phase retention provide more direct reactor evidence than contaminant-specific N_2_O emissions.

### 5.1. Effluent Nitrogen Risk

A key risk pathway is increased effluent nitrogen when nitrogen removal becomes unstable. When anammox activity or nitrite supply is disturbed, ammonium, nitrite, or nitrate can increase in the effluent. Laboratory studies with PFOA, Cu(II), and other toxicants have shown that severe exposure can cause large losses in nitrogen removal [29,33]. However, the concentrations used in many of these studies are much higher than those expected in most wastewaters. These results should therefore be viewed as evidence of possible failure modes rather than as environmental thresholds.

The form of nitrogen released is also important. Nitrite is toxic to aquatic organisms and can indicate an imbalance between nitrite production and consumption [39,40]. In PN/A systems, nitrite may accumulate when anammox bacteria are inhibited more strongly than ammonia-oxidizing bacteria. In PD/A systems, changes in partial denitrifiers can also affect nitrite production. Increased nitrite may further favor N_2_O formation in associated nitrification and denitrification pathways [41,42]. Effluent nitrogen should therefore be treated as both a process indicator and a risk output. Risk assessment should consider changes in individual nitrogen species rather than relying only on total nitrogen removal.

### 5.2. Resistance-Related and Microbial Risk

Contaminant exposure can also change the abundance and distribution of antibiotic resistance genes (ARGs), antibiotic-resistant bacteria (ARB), mobile genetic elements, and other microbial risk factors. These factors can enter anammox systems with the influent, but they may also be retained or selected within dense granules and biofilms [8,9,35]. The environmental concern is therefore not simply whether ARGs or ARB are present, but whether the treatment process changes their abundance, mobility, or release.

Studies with antibiotics have reported clear changes in ARG profiles during exposure [16,23]. The PFBS study also reported enrichment of ARGs, mobile genetic elements, and potential pathogens while nitrogen removal declined [30]. These observations suggest that microbial risk can change even when conventional process indicators provide limited warning. Relative abundance alone, however, does not fully describe this risk. An increase in the relative abundance of an ARG does not necessarily mean that the total ARG load leaving the reactor has increased. Risk assessment should therefore include absolute gene abundance where possible, together with mobile genetic elements, viable resistant bacteria, potential hosts, and the distribution of these factors between water and biomass. This distinction is important because anammox systems can retain microbial material in biomass while also releasing cells or extracellular DNA through effluent solids.

### 5.3. Potential N_2_O Emission Risk

Potential N_2_O emissions are relevant because high nitrogen removal does not necessarily mean low greenhouse-gas release. The core anammox reaction mainly produces N_2_, but practical PN/A and PD/A systems also contain AOB, nitrite-oxidizing bacteria (NOB), denitrifiers, and heterotrophs. Changes in oxygen supply, nitrite concentration, organic carbon, and toxic stress may affect N_2_O production and consumption by these associated populations [41,42].

Direct evidence linking individual emerging contaminants to N_2_O emissions from anammox based reactors remains limited. The main concern is indirect. Contaminant stress can disturb microbial balance, increase nitrite accumulation, or change oxygen and carbon use. These changes may create conditions that favor transient N_2_O peaks during inhibition or recovery. Such peaks may not be detected by occasional gas measurements. Studies that evaluate the environmental effects of contaminant-stressed anammox systems should therefore include direct and sufficiently frequent N_2_O monitoring [43]. At present, this pathway should be considered a potential risk that requires stronger experimental evidence.

### 5.4. Contaminant Accumulation and Release from Retained Biomass

A decrease in dissolved contaminant concentration does not necessarily mean that the contaminant has been destroyed or removed from the treatment system. Granules, biofilms, carriers, and added sorptive materials can retain chemicals, particles, ARGs, and resistant bacteria. This retention can reduce short term exposure in the liquid phase and help maintain nitrogen removal, but it can also increase the contaminant burden in solids. Studies with plastic particles show that substantial accumulation can occur in anammox biomass during long-term exposure [17]. PFOA studies with iron-loaded biochar also show that improved removal from the liquid phase may partly result from transfer to solids rather than degradation [18]. These examples illustrate an important difference between aqueous removal and complete contaminant removal.

Retained contaminants may later leave the reactor with wasted sludge, detached biofilm, lost carrier material, or biomass washout. Sewage sludge is already recognized as a reservoir of antibiotics, ARGs, and ARB [44]. A recent study of granular anammox systems also showed that effluent-released sludge retained nitrogen-transformation potential and raised potential biosafety concerns [45]. These findings indicate that the system boundary should include wasted sludge, detached biomass, and spent carrier materials. A practical balance would track influent and effluent contaminant loads, retained biomass, wasted sludge, detached biofilms, and spent carriers; gaseous nitrogen releases are considered separately.

This pathway is particularly important for risk evaluation because a control measure can improve effluent quality without reducing the total contaminant burden. Sorption to EPS, sludge, biochar, or carriers may protect anammox activity but shift part of the environmental burden from water to solids. The fate and final handling of these solids should therefore be included when treatment performance is evaluated.

**Figure 3:**
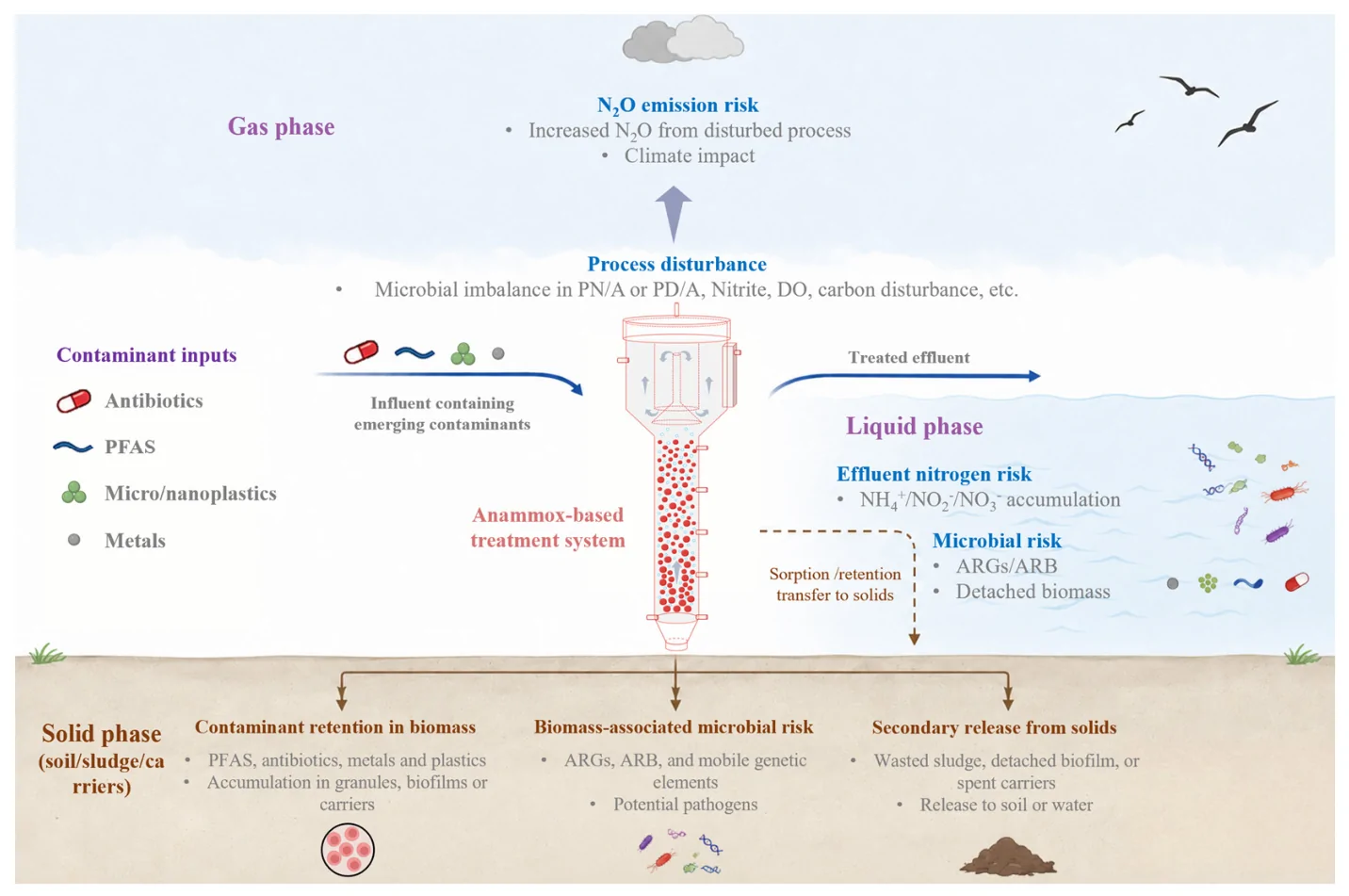
Environmental risk pathways of contaminant-stressed anammox systems across liquid, gas, and solid phases. Contaminant stress can transfer risk to the liquid, gas, and solid phases through effluent nitrogen accumulation, ARGs/ARB and detached biomass, potential N_2_O emissions, contaminant retention in biomass, and secondary release from solids.

The four pathways are linked rather than independent (Figure 3). Nitrogen leakage reflects loss of process function. Microbial changes can affect resistance-related risks and may also influence N_2_O formation. At the same time, contaminant retention can protect reactor performance while increasing the burden in solids. Environmental assessment should therefore examine what leaves or remains in the system through the liquid, gas, and solid phases. Nitrogen removal alone cannot capture these different outcomes. These risk pathways also provide the basis for the monitoring and control strategies discussed in Section 6.

## 6. Monitoring, Early Warning, and Resilience Management

Section 5 identified the main risks of contaminant-stressed anammox systems in the liquid, gas, and solid phases. These risks should be identified before contaminant stress leads to serious process failure or long-term environmental release. Monitoring should therefore extend beyond effluent nitrogen and include changes in biomass condition and risk related outputs. A practical management strategy can be organized into three levels: routine monitoring, early warning and diagnosis, and mitigation and recovery.

### 6.1. Monitoring from Process Performance to Risk Indicators

Routine process indicators provide the first level of monitoring because they show whether nitrogen conversion remains stable. Ammonium, nitrite, nitrate, total nitrogen, COD, alkalinity, pH, dissolved oxygen (DO), oxidation reduction potential (ORP), temperature, free ammonia (FA), and free nitrous acid (FNA) should be monitored according to reactor configuration [2,3,10]. In PN/A and PD/A systems, persistent nitrite accumulation, changes in nitrate production, or unstable pH and ORP profiles can indicate an imbalance between nitrite production and consumption.

Process indicators alone cannot identify the source of the disturbance. Biomass indicators provide a second level of information. SAA, biomass inventory, particle size, settling ability, EPS, granule strength, and membrane integrity can help distinguish biological inhibition from structural damage, poor mass transfer, or biomass loss [10,11,12,13,14,15,35]. Measurements of functional genes can support this diagnosis by revealing changes in anammox metabolism and associated nitrogen-conversion pathways [37,38,42]. For streams containing antibiotics or biocides, selected ARGs and mobile genetic elements can also be included when resistance risk is relevant [8,9].

Risk related indicators form a third level of monitoring. These indicators include N_2_O emissions, contaminant accumulation in biomass, ARGs and ARB in the liquid and solid phases, and the contaminant burden in wasted sludge or spent carriers. These measurements are not required at the same frequency as routine water quality monitoring, but they are important for long-term assessment and for confirming whether risk has been reduced rather than transferred to another phase.

Warning limits should be based on the normal behavior of each reactor. Values reported in batch studies cannot be directly applied to continuously operated systems because biomass retention, acclimation, and exposure history can change reactor tolerance [20,32]. A useful warning limit should therefore be defined relative to a stable operating baseline and supported by more than one indicator. For example, declining SAA together with stable TN removal may indicate hidden loss of cell specific activity, while rising nitrite should be interpreted together with nitrate production, DO, loading, and biomass activity.

### 6.2. Tiered Early Warning and Diagnosis

Early warning becomes more useful when indicators are grouped by their response time. Fast signals include nitrite, DO, pH, ORP, nitrogen loading, and, where available, continuous N_2_O measurements. These variables can show that substrate supply, anammox activity, or microbial balance is becoming unstable before complete process failure occurs.

Biomass indicators usually respond more slowly but provide more information on the cause of the disturbance. SAA, EPS, particle size, settling behavior, microscopy, and membrane integrity can help determine whether the problem is mainly physiological, structural, or related to biomass retention. Community structure, functional genes, ARGs, and contaminant accumulation in biomass are more suitable for longer term diagnosis and for evaluating recovery [10,43].

No warning signal should be interpreted alone. A sustained rise in nitrite can result from anammox inhibition, excessive nitrite production, or poor mass transfer. Stable TN removal together with declining SAA may indicate that a larger biomass inventory is temporarily compensating for lower activity per unit biomass. An increase in EPS may represent a protective response, but a large or persistent change may also indicate particle retention or granule damage. Diagnosis should therefore rely on combinations of process, biomass, and risk indicators. Table 3 links representative warning signals with additional checks and short-term operating responses.

### 6.3. Mitigation and Stepwise Recovery

Mitigation strategies can be grouped into biological, metabolic-regulation, material-based, and process-optimization approaches. Biological approaches include gradual acclimation and, where necessary, bioaugmentation. Gradual acclimation can improve the tolerance of anammox biomass to contaminant exposure [22], while bioaugmentation may help restore activity by introducing stress-tolerant microorganisms [31]. However, acclimation requires time, and the effectiveness of bioaugmentation may depend on the operating conditions.

Metabolic-regulation approaches, such as quorum-sensing-related regulation, may help maintain anammox activity through changes in EPS properties and microbial responses [36]. However, their effectiveness may vary with contaminant type and operating conditions.

Material-based approaches use sorptive or redox-active materials to reduce contaminant exposure and support anammox activity [18,33]. However, these materials may increase costs or transfer contaminants from water to solids. Their benefits should therefore be evaluated beyond nitrogen removal alone.

Process-optimization approaches focus on reducing contaminant loading and maintaining stable operating conditions. Risk control should start before contaminants reach anammox biomass. Equalization, source separation, targeted pretreatment, and control of concentrated side streams can reduce sudden toxic shocks and long-term contaminant loading. Within the reactor, stable nitrite supply, controlled aeration, appropriate hydraulic and solids retention times, and avoidance of sharp changes in salinity, organic loading, FA, and FNA can reduce additional stress [2,3,10,14]. Granules, biofilms, carriers, and membrane systems improve biomass retention, but they can also retain contaminants. Their protective role should therefore be evaluated together with contaminant accumulation.

Recovery after severe inhibition should follow a stepwise process. The first action is to stop, divert, or dilute the toxic input. Operators should then retain as much active biomass as possible and restore nitrite supply at a lower loading rate. SAA and substrate conversion should be checked before loading is increased again [20,22]. This approach reduces the risk of a second shock during early recovery.

Normal effluent quality should not be treated as the end point of recovery. Persistent changes in SAA, functional genes, granule structure, key microbial populations, ARGs, N_2_O emissions, or contaminants retained in solids may indicate incomplete recovery. Severe cases may require longer recovery periods, bioaugmentation, partial biomass replacement, or specific management of contaminated solids.

**Figure 4:**
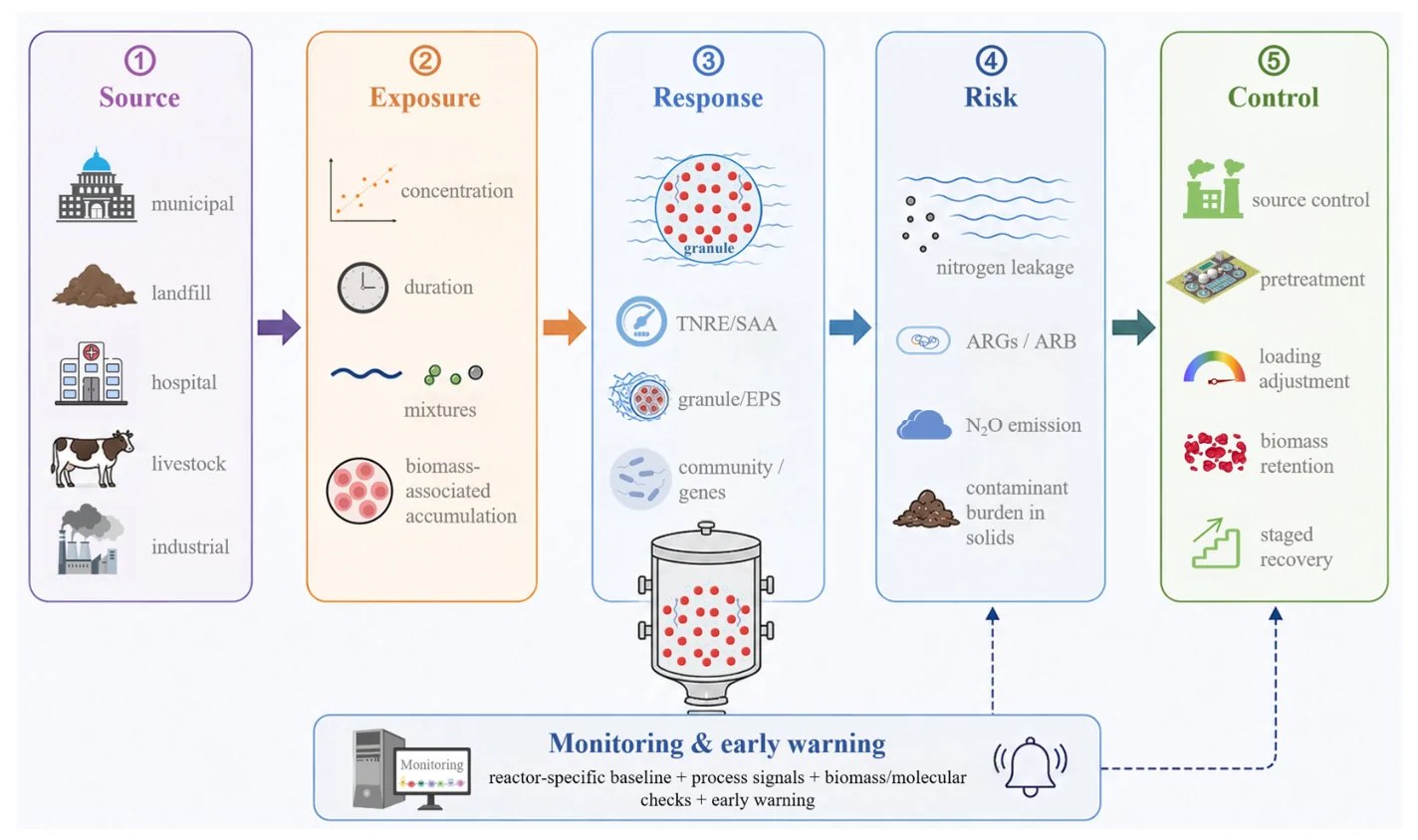
Conceptual schematic of the proposed source-exposure-response-risk-control framework for monitoring and managing contaminant-stressed anammox systems.

Monitoring and management can therefore be integrated into a source-exposure-response-risk-control framework (Figure 4). Source analysis identifies where contaminants enter the system. Exposure assessment describes their concentration, duration, mixtures, and retention in biomass. Monitoring detects changes in process performance and biomass condition. Risk assessment follows outputs to the liquid, gas, and solid phases. Control measures then reduce exposure, limit risk transfer, and support recovery. The results of monitoring should feed back into source control and operating decisions.

This framework links routine process control with environmental risk management. Its main purpose is not to define a single warning threshold for all anammox systems, but to support reactor specific decisions based on baseline conditions, multiple indicators, and recovery at both process and biomass levels.

## 7. Knowledge Gaps and Practical Priorities

Current evidence shows clear effects of contaminant stress on anammox systems, but practical use is still limited by simplified exposure conditions, inconsistent reporting, and limited field validation [10,11,12,13,14,15]. Future work should therefore focus on evidence that is more realistic, comparable, and useful for operation.

### 7.1. More Realistic Exposure Conditions and Contaminant Mixtures

Most studies still use single compounds, synthetic wastewater, and short exposure periods, whereas real systems face long-term mixtures under changing temperature, salinity, organic carbon, and loading [4,5,6,7,8,9,10,11,12,13,14,15]. Future studies should use exposure profiles that represent municipal wastewater, sidestream reject water, landfill leachate, hospital and pharmaceutical wastewater, livestock and aquaculture wastewater, and industrial high-ammonium streams.

Long-term low-dose mixtures deserve particular attention. Recent work has begun to examine combined PFAS and quaternary ammonium compound stress in nitrification-anammox biofilms [21], but mixture studies remain limited. Experiments should distinguish immediate inhibition, adaptation during continuous exposure, and recovery after contaminant removal.

### 7.2. Comparable Reporting and Stronger Mechanistic Evidence

Cross-study comparison requires consistent reporting of contaminant concentrations in liquid and, where relevant, biomass; particle properties; biomass source and form; reactor configuration and major operating conditions; exposure duration; nitrogen-removal performance and SAA; and post-exposure recovery [10,11,12,13,14,15].

Mechanistic conclusions should match the strength of the evidence. Intracellular contaminant measurements, enzyme activity, cell damage, transcripts, or proteins provide stronger support than community correlations alone. Co-occurrence networks may suggest microbial associations but do not confirm host identity or horizontal gene transfer, while molecular docking cannot prove inhibition in living cells. High-dose experiments can reveal hazards and failure modes but should not be interpreted as representing typical wastewater exposure or as defining operating limits.

### 7.3. Pilot-Scale Validation and Practical Use

Laboratory findings need validation under pilot- and full-scale conditions, where temperature, hydraulic loading, organic carbon, salinity, and contaminant mixtures vary together. Field studies should combine routine process monitoring with selected biomass, molecular, N_2_O, and solids-associated indicators [2,4,43,45]. Well-described low-dose or no-effect cases are also valuable for defining reactor baselines.

Warning indicators and control actions should be tested during continuous operation before they are used as operating rules. Mitigation strategies should also be assessed beyond nitrogen removal, with benefits weighed against costs, operational complexity, secondary toxicity, ARGs, N_2_O emissions, and contaminant transfer to sludge or carriers [2,10,43,44].

Overall, future research should focus on realistic mixtures, comparable reporting, stronger mechanistic evidence, and pilot-scale validation. These priorities would help translate laboratory toxicity data into reactor-specific guidance for environmental risk monitoring and control.

## 8. Conclusions

Contaminant stress can affect anammox systems at both process and microbial levels, with responses depending on contaminant type, concentration, exposure duration, biomass form, and exposure history. Short-term tolerance does not necessarily indicate long-term stability, because contaminants retained in biomass may accumulate and cause delayed inhibition, while acclimation can maintain nitrogen removal even when cell-specific activity or microbial characteristics have already changed. Across studies, these effects generally involve contaminant retention in EPS and biomass, structural and cellular stress, disruption of redox processes and core anammox metabolism, and shifts in microbial interactions. These responses can further translate into environmental risks through ammonium or nitrite leakage, resistance-related changes, potential N_2_O emissions, and contaminant retention or release from sludge and biofilms. Building on these findings, this review proposes a source-exposure-response-risk-control framework to link contaminant exposure and anammox responses with environmental risk assessment and management. Anammox systems under contaminant stress should therefore be evaluated not only by nitrogen removal performance but also by microbial responses, recovery status, and risk transfer across liquid, gas, and solid phases. Because the evidence remains mainly laboratory-based and direct environmental risk measurements are limited, the proposed framework is better used as a qualitative guide for reactor-specific monitoring.

## Figures and Tables

**Table 1:** Qualitative comparison of contaminant exposure in anammox configurations and biomass forms.

Configuration or Biomass Form	Typical Exposure Consideration
Sidestream PN/A	Ammonium-rich reject water; high ammonium concentration, salinity, and organic matter may influence contaminant exposure and microbial responses.
Mainstream PN/A	Low-strength municipal wastewater; low ammonium concentration, variable temperature, and organic carbon may affect responses to trace contaminants.
Single-stage PN/A	Ammonia-oxidizing and anammox bacteria share the same influent, although their local exposure may differ within granules or biofilms.
Two-stage PN/A	The anammox reactor receives effluent from the upstream nitritation stage, so contaminant exposure may differ between the two stages.
PD/A	Contaminants may affect both partial denitrifiers and anammox bacteria, potentially changing nitrite availability.
Granular sludge	EPS and internal concentration gradients may reduce immediate cellular exposure but increase contaminant retention within granules.
Carrier-based biofilms	Biomass retention and diffusion gradients within attached biofilms may influence local exposure and contaminant contact time.
Membrane biofilms	Transport through biofilms and, where relevant, membranes may create concentration gradients that affect local exposure.

Note: These configurations and biomass forms are not mutually exclusive. The comparison describes possible exposure differences rather than measured toxicity rankings or universal operating thresholds.

**Table 2:** Representative reported responses of anammox systems to contaminants and co-stressors.

Contaminant	System and Exposure	Main Reported Response	Key Pattern
OTC	Granule reactor; 2 mg/L, 3 weeks	SAA reduced 81.3%; heme c reduced 50.1%; *hzs* abundance reduced 29.7%	Strong chronic inhibition [23]
SDZ vs. CTC	Enriched consortium; SDZ 10 mg/L, CTC 1 mg/L	Stable under SDZ; CTC inhibition did not recover	Compound-specific response [16]
SDZ	Repeated shocks vs. stepwise loading; up to 50 mg/L	Repeated shocks caused cumulative decline; stepwise loading kept TNRE > 85%	Exposure history changed tolerance [22]
PET-MPs	Anammox granular sludge; 0.1–1.0 g/L	Little effect at 0.1–0.2 g/L; SAA reduced by 16.2% at 1.0 g/L	Dose-dependent response [24]
PET-MPs	~300 µm; 200 mg/L; long-term laboratory reactor exposure	TNRE declined; recovered to approximately 87% of its initial value after three months	Long-term inhibition and recovery [19]
PS-NPs	1–2 mg/L; 225 d laboratory EGSB reactor	At day 225, ammonium- and nitrite-based SAA were 44.5% and 57.6% below day 100; strong inhibition at 118 mg/g VSS in biomass	Accumulation-related inhibition [17]
LDPE/PP MPs	~0.5 and 4.75 mm; 0.5 g/L; laboratory anammox tests	Little direct inhibition; 31 and 37 compounds were tentatively identified in LDPE and PP leachates, respectively	Particle and leachate effects differ [28]
PE-MPs	~30 µm; 0.05–1.0 g/L; 40 d batch study	Higher concentrations reduced EPS content and SAA, with SAA decreasing by up to approximately 20%	Biomass responses to particle stress [26]
PVC/PBS MPs	0.1 and 0.5 g/L; 40 d laboratory study	At 0.5 g/L, average ammonium removal was 93.3% under PVC and 99.0% under PBS, compared with 99.5% in the control	Polymer-dependent response [27]
DBP	Separately dosed DBP; granular biomass; 100 mg/L; 5–24 h	Inhibition decreased from 29.4% at 5 h to 3.3% at 24 h	Transient chemical effect [28]
PFOA	Anaerobic baffled reactor; 5–50 mg/L	Ammonium removal 93.90% to 77.81%; nitrite removal 98.60% to 77.96%	High-dose inhibition [29]
PFBS	SBR; 0, 200 ng/L, 100 μg/L, and 50 mg/L	TN removal 95.60 ± 0.85% to 85.89 ± 0.93% at the highest dose; *hzs* abundance reduced 54.65%; *hdh* abundance reduced 57.32%	Performance and gene changes [30]
TCC	Long-term reactor; 50 vs. 200 μg/L	Little effect at 50 μg/L; inhibition and community change at 200 μg/L	Dose-dependent biocide response [31]
Ni(II)	Batch: 0–100 mg/L; UASB: 10 mg/L, 150 d	At 1 mg/L, Ni(II) increased SAA [20]; batch IC_50_ values were 83.86 mg/L [20] and 14.64 mg/L [32]; after acclimation, the UASB reached approximately 93% nitrogen removal [32]	Batch and long-term responses differ [20,32]
Cu(II)	Continuous reactor; 5 mg/L	Nitrogen removal efficiency decreased from 99.0% to 25.48% in the Cu(II)-exposed reactor without added biochar	Severe inhibition [33]
ZnO NPs	Batch/long term; 200 mg/L acute, 100 mg/L long term	About 80% inhibition under acute exposure to 200 mg/L; reactor performance recovered within 120 d after long-term exposure	Acute and chronic responses differ [34]

Notes: SAA, specific anammox activity; TNRE, total nitrogen removal efficiency; EPS, extracellular polymeric substances; VSS, volatile suspended solids; UASB, upflow anaerobic sludge blanket; EGSB, expanded granular sludge bed; SBR, sequencing batch reactor; MPs, microplastics; PS-NPs, polystyrene nanoplastics; ZnO NPs, zinc oxide nanoparticles; OTC, oxytetracycline; SDZ, sulfadiazine; CTC, chlortetracycline; PET, polyethylene terephthalate; PS, polystyrene; LDPE, low-density polyethylene; PP, polypropylene; PE, polyethylene; PVC, polyvinyl chloride; PBS, polybutylene succinate; DBP, dibutyl phthalate; PFOA, perfluorooctanoic acid; PFBS, perfluorobutane sulfonate; TCC, triclocarban. Batch IC_50_ values describe toxicity under the specific test conditions and should not be used as engineering thresholds. High concentrations used in some laboratory tests are intended for toxicity assessment and should not be taken as typical concentrations in wastewater. Reactor temperature, nitrogen loading, concentration basis, and recovery conditions are not uniformly specified in these summaries and should not be assumed equivalent.

**Table 3:** Illustrative warning signals, confirmatory checks, and reactor-specific operating responses in contaminant-stressed anammox systems.

Warning Signal	Possible Interpretation	Confirmatory Check	Suggested Operating Response
Sustained nitrite increase	Anammox inhibition, excess nitrite production, or poor mass transfer	SAA; nitrate production; AOB/anammox activity; DO; nitrogen loading	Reduce loading; isolate or dilute the toxic input; restore stable nitrite supply and aeration
TNRE stable but SAA declines	Biomass growth or retention is masking lower cell-specific activity	Biomass inventory; EPS; *hzs*/*hdh*; contaminant mass in biomass	Hold the current loading and follow biomass activity before further load increase
Rapid EPS or granule change	Structural stress, sorption, or particle accumulation	EPS composition; particle size; settling; microscopy; biomass-associated contaminant	Reduce the shock and protect biomass retention while identifying the main stressor
ARG or *intI1* increase	Resistance selection or increased mobility of resistance genes	Absolute gene copies; mobile elements; possible hosts; viable ARB	Strengthen source control and review antibiotic or biocide pretreatment and sludge handling
N_2_O peak	Microbial imbalance, nitrite stress, or carbon/oxygen mismatch	DO; nitrite; COD; *nir*/*nor*/*nos* signals; gas-flow measurement	Rebalance DO, nitrite, loading, and organic carbon
High contaminant burden in sludge or carriers	Retention or sorption rather than complete removal	Water-solid mass balance; leachability; carrier loss; wasted sludge mass	Manage contaminated solids or spent materials and avoid treating sorption as complete removal

Notes: SAA, specific anammox activity; TNRE, total nitrogen removal efficiency; EPS, extracellular polymeric substances; AOB, ammonia-oxidizing bacteria; DO, dissolved oxygen; ARG, antibiotic resistance gene; ARB, antibiotic-resistant bacteria. The listed signals and responses are diagnostic examples rather than universal operating rules and should be interpreted against reactor-specific baselines.

## Data Availability

No new data were created or analyzed in this study. Data sharing is not applicable to this article.
